# Use of zeolite to neutralise nickel in a soil environment

**DOI:** 10.1007/s10661-017-6427-z

**Published:** 2017-12-30

**Authors:** Edyta Boros-Lajszner, Jadwiga Wyszkowska, Jan Kucharski

**Affiliations:** University of Warmia and Mazury in Olsztyn, Plac Łódzki 3, 10-727 Olsztyn, Poland

**Keywords:** Zeolite, Soil, Nickel, Soil enzymes, Plant

## Abstract

Nickel is a heavy metal which is a stable soil pollutant which is difficult to remediate. An attempt to reduce its impact on the environment can be made by changing its solubility. The right level of hydrogen ions and the content of mineral and organic colloids are crucial in this regard. Therefore, methods to neutralise heavy metals in soil are sought. There are no reports in the literature on the possibility of using minerals in the detoxication of a soil environment contaminated with metals. It is important to fill the gap in research on the effect of zeolites on the microbiological, biochemical and physicochemical properties of soils under pressure from heavy metals. Therefore, a pot experiment was conducted on two soils which examined the effect of various levels of contamination of soil with nickel on the activity of soil enzymes, physical and chemical properties and growth and development of plants. An alleviating effect of zeolite Bio.Zeo.S.01 on the negative impact of nickel on the soil and a plant (oats) was examined. The enzyme activity and the oat yield were found to be significantly and negatively affected by an excess of nickel in the soil, regardless of the soil type. The metal was accumulated more in the oat roots than in the above-ground parts. An addition of zeolite decreased the level of accumulation of nickel in oats grown only on sandy-silty loam. Zeolite Bio.Zeo.S.01 used in the study only slightly alleviated the negative effect of nickel on the biochemical properties of soil. Therefore, its usability in the remediation of soil contaminated with nickel is small.

## Introduction

In recent years, zeolites—both natural and synthetic—have attracted researchers’ interest as agents to remove contaminants and for prevention. Owing to their exchange capability and selectivity towards inorganic cations (zinc, lead, cadmium, copper, nickel), anions, as well as organic molecules, such as pesticides and phenols, they can remove many types of contaminants at the same time (Panuccio et al. 2008; Wyszkowski, Sivitskaya 2014).

Zeolites are crystalline, hydrated aluminosilicates of such metals as calcium, magnesium, potassium, sodium, strontium and barium. Like major rock-forming minerals, zeolites have a specific skeletal structure. Primary elements of a zeolite structure include tetrahedrons of aluminium (AlO_4_^−^) and silicon (SiO_4_), where an aluminium or silicon atom is surrounded by four oxygen atoms. Connected tetrahedrons make polyhedrons, i.e. secondary structural units (Erdem et al. 2004; Oren, Kaya 2006). According to Maicaneanu et al. (2013), zeolites have catalytic properties, they enable processes of adsorption and desorption, as well as ion exchange. There are two groups of zeolites: natural and synthetic ones. The group of natural zeolites comprises ca. 70 minerals, whereas there are ca. 100 synthetic zeolites, including Clinoptilolite—(Na_4_K_4_)(Al_8_Si_4_O_96_) 24H_2_O—which is a zeolite of the heulandite group, with a Si-to-Al ratio of 4–5.5—and is the most common natural zeolite. It has a number of specific physical and chemical features, i.e. large sorptive and ion-exchange capacity, selectivity, molecular sieve capacity, catalytic activity (especially in the hydrogenated form) and structural thermostability at high temperatures (700 °C) (Leung et al. 2006).

Because of the range of exceptional physical and chemical features, zeolites are applied in many areas of human activity: industrial technologies, medicine, pharmacy, water and wastewater treatment, ecology, agriculture, soil environment protection (Rehakova et al. 2004). The application of zeolites in the detoxication of soils contaminated with heavy metals is of particular importance.

Since there have been no reports in the literature on the use of minerals in the detoxication of a soil environment contaminated with heavy metals, there was a need for such a study on the application of various zeolites for detoxication, since they have the ability to exchange ions with a given mineral and its chemical properties. This feature is referred to as the cation exchange capacity and it depends mainly on the SiO_2_/Al_2_O_3_ ratio in a zeolite (Ursini et al. 2006). Jamil et al. (2010) points out that—apart from various applications—zeolites exhibit extremely strong affinity to heavy metals. Studies on the effectiveness of binding heavy metals by various zeolites can be found in the literature (Chao and Chen 2012; Seliman and Borai 2011). Unfortunately, these authors did not examine the effect of zeolites on the biological properties of soils. This is why it was important to fill the gap in research on the effect of zeolites on the biochemical and physicochemical properties of soils and yield of crops under pressure from heavy metals. Therefore, research was conducted to determine the possibility of alleviating the effect of nickel on the activity of soil enzymes and on oat yield by introducing zeolite Bio.Zeo.S.01 to soil.

## Materials and methods

### Soil

Soil for the experiment was sampled from the surface layers (from the depth of 0–20 cm) in the field at the Teaching and Research Station in Tomaszkowo in the Olsztyn Lake District, with proper brown soil and leached brown soil dominating (north-east of Poland, 53.7161^o^ N, 20.4167° E). The experiment was set up in a vegetation hall owned by the University of Warmia and Mazury. The soil on which the experiment was conducted is classified as loamy sand and sandy-silty loam. These soils in a natural condition were classified as Eutric Cambisols based on the Word Reference Base for Soil Resources (2014). The soil characteristics are provided in Table 1.

**Table 1:** Some physicochemical properties of the soils used in the experiment

Type of soil	Granulometric composition (mm)			C_org_ (g kg^−1^)	N_total_	pH_KCl_	HAC	EBC	CEC	BS %
	< 0.002	0.020–0.050	0.050–2.000				(mmol^(+)^ kg^−1^ of soil)			
ls	2.27	25.31	72.42	9.44	0.71	6.80	11.38	69.00	80.38	85.84
sl	2.88	27.71	69.41	14.30	0.98	7.00	6.40	165.90	172.30	96.29

ls loamy sand, sl silty-sandy loam, C_org_ total organic carbon, N_total_ total nitrogen, HAC hydrolytic acidity, EBC total exchangeable cations, CEC total exchange capacity of soil, BS basic cations saturation ratio in soil

### Setting up the experiment

The experiment was set up by putting 3.5 kg of soil in each of the plastic pots. The factors under study included the following:

- the type of soil: loamy sand, sandy-silty loam,
- a dose of nickel as chloride (NiCl_2_·7H_2_O), mg of Ni^2+^ kg^−1^ of soil 0, 100, 200 and 400,
- an addition of zeolite Bio.Zeo.S.01 in milligram per kilogram of soil 0 and10,
- the time of soil incubation, days 25 and 50.

Oats (*Avena sativa* L.) of the Kasztan variety were used as the experimental crop (12 plants per pot). Fertilisation with macro- and micronutrients at a constant level was applied in all of the pots. Moisture content at 50% of the capillary water capacity was maintained throughout the experiment. Oats were harvested at the BBCH 52 phase—20% of inflorescence emerged. The above-ground parts were dried at 70 °C and their weight was determined.

### Characteristics of the zeolite

The zeolite used in the experiment was provided by the BioDrain company in Rzeszów. This sorbent is a natural zeolite. Zeolite Bio.Zeo.S.01 was applied in the experiment at 10 g kg^−1^ of soil, with the following composition (% of oxides): SiO_2_—70.6, Al_2_O_3_—12.32, Fe_2_O_3_—1.48, TiO_2_—0.71, MnO_2_—0.02, CaO—3.42, MgO—0.96, K_2_O—2.83, Na_2_O—0.68, the content of pure clinoptylolite 60.

### Biochemical and physicochemical analyses

Twice during the growing season for oats (day 25 and 50), the activity of dehydrogenases [EC 1.1] was determined by the Öhlinger method (1996), the activity of catalase [EC1.11.1.6] and urease [EC 3.5.1.5] by the Alef and Nannipieri method (1998) and of acid phosphatase [EC 3.1.3.2], alkaline phosphatase [EC 3.1.3.1], ß-glucosidase [EC 3.2.1.21] and arylsulfatase [EC 3.1.6.1] as per the procedures described by Alef et al. (1998). The following chemical compounds were used as substrates to determine the activity of dehydrogenases: 2,3,5-triphenyltetrazolium chloride, of catalase—hydrogen peroxide, of urease—urea, of acid and alkaline phosphatase—4-nitrophenylphosphate disodium, of ß-glucosidase—p-nitrophenyl-B-D-glucopyranoside and of arylsulfatase—potassium 4-nitrophenyl sulphate. The activity of individual enzymes was expressed in the following units: dehydrogenases—μmol TFF kg^−1^ d.m. h^−1^, catalase—mol O_2_ kg^−1^ d.m. h^−1^, urease—mmol N-NH_4_ kg^−1^ d.m. h^−1^, acid and alkaline phosphatase, ß-glucosidase and arylsulfatase—mmol PNP kg ^−1^ d.m. h^−1^. Enzymatic assays were performed on a Perkin-Elmer Lambda 25 spectrophotometer, except determination of activity of catalase. Activity of dehydrogenase was determined at *λ* = 485 nm, urease, acid and alkaline phosphatase at *λ* = 410 nm; ß-glucosidase at *λ* = 400 nm and arylsulfatase at *λ* = 420 nm. Indexes of the impact of nickel (IF_Ni_) and zeolite (IF_z_) on the activity of soil enzymes were calculated from the following formulae (Kaczyńska et al. 2015):

$$ {\mathrm{IF}}_{\mathrm{Ni}}=\frac{{\mathrm{A}}_{\mathrm{Ni}}}{{\mathrm{A}}_0} $$

where:

IF_Ni_: index of nickel impact,
A_Ni_: activity of enzymes in soil contaminated with nickel,
A_0_: activity of enzymes in uncontaminated soil.

$$ {\mathrm{IF}}_{\mathrm{z}}=\frac{{\mathrm{A}}_{\mathrm{z}}}{\mathrm{A}} $$

where:

IF_Z_: index of zeolite impact,
A_Z_: activity of enzymes in soil with an addition of zeolite,
A: activity of enzymes soil without an addition of zeolite.

Moreover, a bioconcentration index (BAC) was calculated, taking into account the content of nickel in the above-ground parts of the oats and in soil.

$$ \mathrm{BAC}=\frac{{\mathrm{C}}_{\mathrm{A}}}{{\mathrm{C}}_{\mathrm{S}}} $$

where:

C_A_: nickel content in above-ground parts,
C_S_: nickel content in roots.

Nickel absorption by oats and its distribution in above-ground parts of plants was also calculated.

The granulometric composition of the soil was determined with a Mastersizer 2000 laser particle-sizing instrument. After the harvest (day 50) of oats, the following physical and chemical characteristics of soil were determined: pH of soil—potentiometrically in aqueous solution of KCl at 1 mol dm^−3^, hydrolytic acidity (HAC), total exchangeable basic cations (EBC) according to Kappen (Carter 1993), content of organic carbon (C_org_)—by Turin’s method (Nelson and Sommers 1996), total nitrogen by Kjeldahl’s method (Nelson and Sommers 1996). Considering hydrolytic acidity (HAC) and total exchangeable basic cations (EBC), the total cation exchange capacity of soil (CEC) and soil saturation with basic cations was calculated (BS). The content of nickel in soil was determined by flame atomic absorption spectrometry (FAAS) following mineralisation in aqua regia, on an AAS 30 apparatus as per PN-ISO 11047: 2001P. The content of Ni in the plant material was determined by atomic absorption spectroscopy (Perkin-Elmer Analyst 800) with a graphite cuvette with electrothermal heating, at the wavelength of *λ* = 232 nm as per PN-ISO 11047: 2001P.

### Statistical analysis

The results were analysed statistically with an ANOVA by means of the Statistica 12.5 software (Statsoft, Inc, Statistica 2015). Multi-dimensional exploration techniques were used to analyse the activity of enzymes in soil contaminated with nickel and with an addition of zeolite by means of the principal component analysis (PCA). The *η*^2^ coefficient of percentile variability of all the variables being analysed was then determined by analysis of variance—ANOVA.

## Results and discussion

The experiment found a negative effect of soil contamination with nickel on the activity of enzymes under study. The activity of dehydrogenases was largely determined by the level of contamination of soil with nickel and the time of soil incubation; that of urease, acid and alkaline phosphatase—by the type of soil and the dose of nickel; the activity of catalase and β-glucosidase—by the type of soil, and the activity of arylsulfatase—by the type of soil and the duration of the experiment (Table 2).

**Table 2:** Percentage of factors of observed variability *η*^2^

Variable factors	Deh	Cat	Ure	Pac	Pal	Glu	Aryl
Dose Ni^2+^ (a)	37.18	17.45	43.62	36.48	33.14	7.86	2.89
Type of soil (b)	9.51	55.90	33.88	45.44	45.02	76.63	60.92
Addition of zeolite (c)	2.34	2.87	3.91	2.26	6.41	1.38	6.21
Time of soil incubation (d)	37.88	4.82	0.41	0.57	7.00	0.80	22.92
a b	2.56	0.12	7.40	1.88	0.69	1.89	0.22
a c	0.09	0.67	0.79	0.39	0.70	0.53	0.09
a d	1.31	6.51	2.84	1.86	0.33	0.72	0.32
b c	0.11	0.53	0.18	0.08	0.19	0.16	0.83
b d	3.35	0.50	1.55	0.20	2.02	0.15	0.51
c d	0.06	0.00	0.17	2.25	0.10	4.18	3.75
a b c	1.37	0.36	0.41	0.27	1.84	0.91	0.04
a b d	1.57	0.45	1.54	0.72	0.16	0.30	0.06
a c d	1.12	0.88	1.36	0.25	0.19	2.09	0.23
b c d	0.40	0.08	0.23	3.27	1.31	0.11	0.60
a b c d	0.42	1.63	0.42	1.37	0.11	2.05	0.20
Error	0.73	7.23	0.81	1.56	0.11	0.22	0.20

Deh, dehydrogenases; Cat catalase; Ure urease; Pac acid phosphatase; Pal alkaline phosphatase; Glu ß-glucosidase; Aryl arylsulfatase

**Fig. 1:**
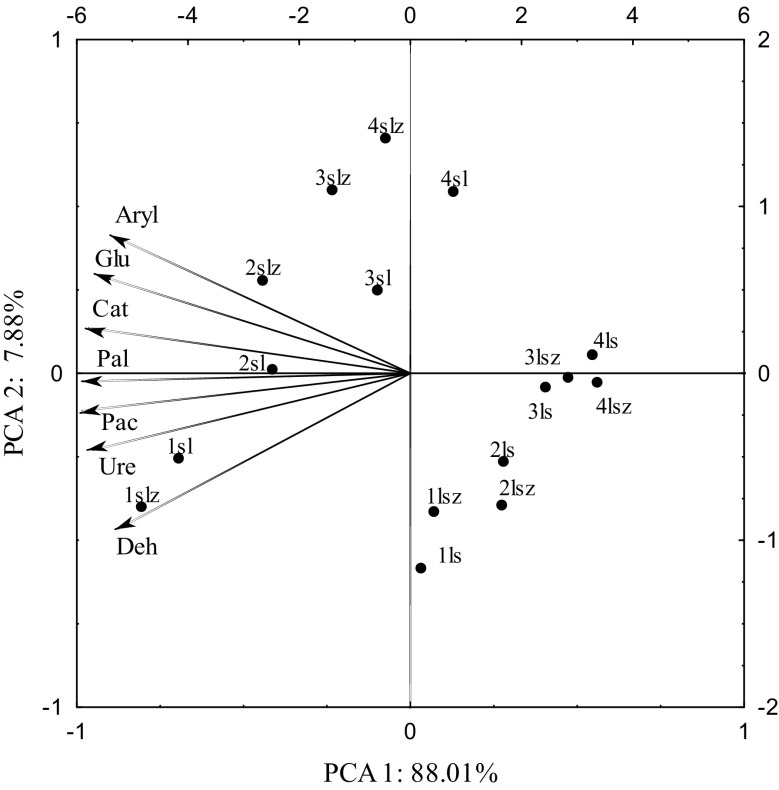
Activity of enzymes in soil contaminated with nickel with an addition of zeolite. Dose Ni^2+^ 1 kg^−1^ d.m. of soil 1, 0 mg; 2, 100 mg; 3, 200 mg; 4, 400 mg; z, soil with an addition of zeolite; ls, loamy sand, sl, silty-sandy loam; Deh, dehydrogenases; Cat, catalase; Ure, urease; Pac, acid phosphatise; Pal, alkaline phosphatise; Glu, ß-glucosidase; Aryl, arylsulfatase

**Fig. 2:**
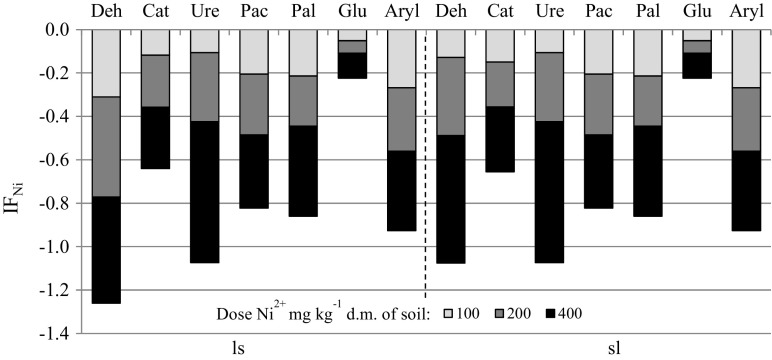
Index of the effect of nickel (IF_Ni_) on enzyme activity. ls, loamy sand; sl, silty-sandy loam; Deh, dehydrogenases; Cat catalase; Ure urease; Pac, acid phosphatise; Pal alkaline phosphatise; Glu, ß-glucosidase; Aryl arylsulfatase

The distribution of vectors which describe the activity of enzymes presented with the principal component analysis (PCA) indicates a significant relationship between the enzyme activity and the dose of the heavy metal applied (Fig. 1). Nickel applied at 100, 200 and 400 mg Ni^2+^ kg^−1^ d.m. of soil inhibited soil enzymes. This is shown by the distribution of cases on a plane. Dehydrogenases, urease, acid phosphatase and alkali phosphatase were negatively correlated with both the first and the second principal component. The vectors representing the original variables are arranged in a similar manner. Their arrangement proves that the above-mentioned enzymes responded in a similar manner to the contamination with Ni^2+^ (Table 3). Therefore, alkaline phosphatase, acid phosphatase, urease and dehydrogenases made one group, but they were more sensitive to a negative effect of nickel, which had a toxic effect on soil enzymes, decreasing their activity with increasing contamination despite applying zeolite Bio.Zeo.S.01 to the soil. Arylsulfatase, β-glucosidase and catalase reacted similarly to soil contamination with nickel, which is confirmed by the close position of the vectors of these enzymes. The above-mentioned enzymes were negatively correlated with the first principal component and positively correlated with the second principal component. The negative effect of nickel on soil enzymes is reflected in the index of the effect of nickel (IF_Ni_) on biochemical activity. Considering the values of the index (IF_Ni_), the analysed enzymes were arranged in the following order (from the most sensitive to Ni^2+^ to the least sensitive): dehydrogenases > urease > arylsulfatase > alkaline phosphatase > acid phosphatase > catalase > β-glucosidase (Fig. 2). A negative effect of the metal was apparent in soil samples containing the lowest level of nickel (100 mg Ni^2+^ kg^−1^ d.m.) and it intensified with increasing contamination. This is confirmed by the findings of a study conducted by Wyszkowska et al. (2008), who observed decreased activity of dehydrogenases following the application of nickel at 100 mg Ni^2+^ kg^−1^ d.m. of soil; the activity of the other enzymes: urease, acid phosphatase and alkaline phosphatase decreased after the application of 200 and 400 mg Ni^2+^ kg^−1^ d.m. of soil. The findings of a study conducted by Boros et al. (2011) also indicate a negative impact of nickel on enzymatic activity in soil. An adverse effect of heavy metals, including nickel, depended on the level of soil contamination with them. This is because nickel can stimulate enzyme activity at low levels (from 25 to 75 mg Ni^2+^ kg^−1^ d.m. of soil), but when the acceptable levels (150 mg Ni^2+^ kg^−1^ d.m. of soil) are exceeded, it becomes a typical inhibitor of soil enzymes and microorganisms (Kucharski et al. 2011; Wyszkowska et al. 2013).

**Table 3:** Correlations of enzymatic activity with PCA 1 and PCA 2 was inserted

Enzymes	PCA 1	PCA 2
Dehydrogenases	− 0.875	− 0.465
Catalase	− 0.963	0.136
Urease	− 0.951	− 0.230
Acid phosphatase	− 0.983	− 0.112
Alkaline phosphatase	− 0.982	− 0.024
ß-glucosidase	− 0.924	0.291
Arylsulfatase	− 0.882	0.408

**Fig. 3:**
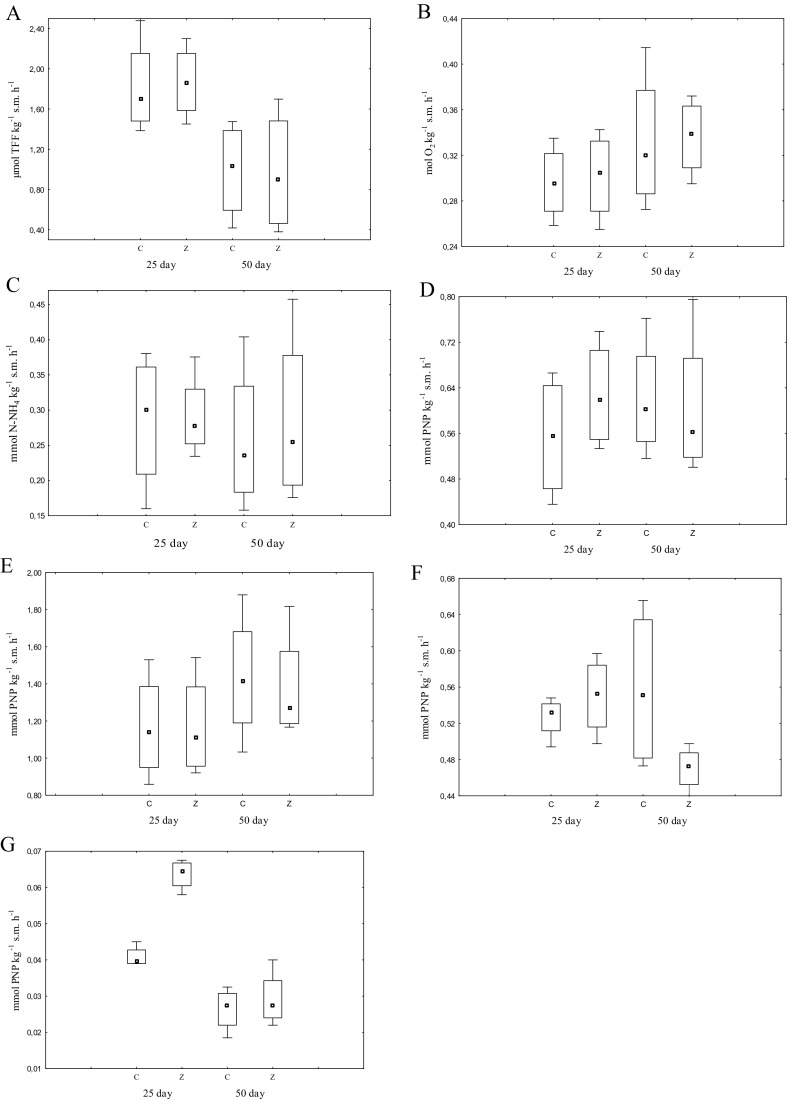
Activity of enzymes in soil contaminated with nickel depending on the time of soil incubation. C, control; Z, soil with an addition of zeolite; 25 day; 50 day—Time of soil incubation; **a** dehydrogenases; **b** catalase; **c** urease; **d** acid phosphatise; **e** alkaline phosphatise; **f** ß-glucosidase; **g** arylsulfatase

Biochemical activity of the soil significantly depended not only on the extent of soil contamination with nickel, but also on the time of exposure of soil microbiome to the metal (Fig. 3). The activity of dehydrogenases, urease and arylsulfatase on day 25 of the experiment was higher in all pots than a month later. It was the reverse for catalase and alkaline phosphatase, because their activity on day 50 was higher than on day 25, regardless of the type of soil and addition of zeolite. The duration of the experiment had little effect on the activity of acid phosphatase and β-glucosidase. On average, zeolite Bio.Zeo.S.01 increased the activity of dehydrogenases, catalase, acid phosphatase, β-glucosidase and arylsulfatase on day 25 of the experiment and catalase and urease on day 50, regardless of the type of soil and the level of contamination with nickel. The zeolite decreased the activity of dehydrogenases, acid phosphatase, alkaline phosphatase, β-glucosidase on day 50.

**Fig. 4:**
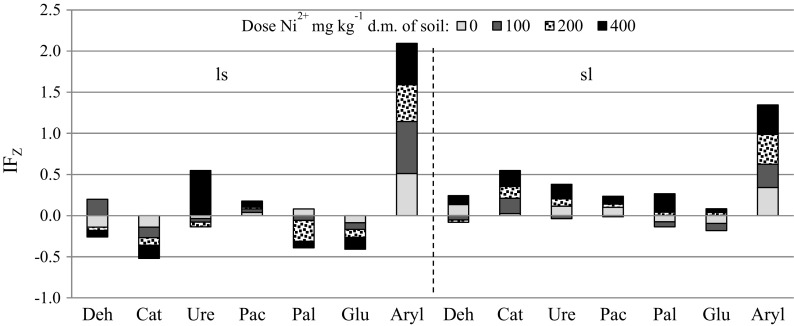
Index of effect of zeolite (IF_Z_) on the activity of enzymes in soil contaminated with nickel. ls, loamy sand; sl, silty-sandy loam; Deh, dehydrogenases, Cat catalase, Ure urease, Pac acid phosphatise; Pal alkaline phosphatise; Glu ß-glucosidase; Aryl arylsulfatase

An effect of zeolite Bio.Zeo.S.01 on enzymatic activity of soil is reflected very well in the index of zeolite effect (IF_Z_). An analysis of the effect of zeolite on the biological activity of soil, regardless of the duration of the experiment, shows that a negative effect of nickel on soil enzymes was alleviated with the sorbent added to a soil only to a small extent (Fig. 4). The zeolite alleviated the negative effect of nickel, both in loamy sand and in sandy-silty loam and in the case of arylsulfatase and acid phosphatase. An analysis of the findings in regard to the soil type showed that the effect of zeolite was better in sandy-silty loam than in loamy sand. It was visible in soil samples contaminated with the highest dose of the metal (400 mg Ni^2+^ kg^−1^ d.m. of soil). To date, studies have focused on the use of zeolites to immobilise heavy metals in soil. Chao and Chen (2012) focused on the capabilities of modified and non-modified zeolites in binding cations: copper, zinc, nickel, lead, cadmium, and anions: dichromate and permanganate. Seliman and Borai (2011) examined the properties of two minerals (chabazite, mordenite) and proposed using them as a specific barrier to immobilise heavy metals (Zn, Co, Ni). Furthermore, Ismael (2010) determined the sorptive effectiveness of zinc by testing a synthetic zeolite obtained from kaolin (zeolite X). Other studies have focused mainly on the use of zeolites (clinoptilolite, bentonite) to purify water (Jovanovic et al. 2012) or wastewater (Shi et al. 2013). The literature (Jamil et al. 2010; Seliman and Borai 2011; Chao and Chen 2012) provides reports on diverse applications of zeolites, especially on their strong affinity in binding heavy metals. Zeolites with monovalent ions, such as sodium, are more effective in ion exchange compared to bivalent ions (calcium, magnesium). The authors’ own studies did not find Zeolite Bio.Zeo.S.01 to neutralise the negative impact of nickel on biochemical activity of soil. This can be attributed to the selectivity of clinoptilolite in absorbing heavy metal ions from the environment: Pb > Cd > Cs > Cu(II) > Co(II) > Cr(III) > Zn > Ni(II) > Hg(II) (Tomczak and Kamiński 2012). As noted earlier, one of the major features of zeolites is the ability to exchange ions with respect to a given mineral and its chemical properties. This feature is referred to as the ion exchange capacity and it depends mainly on the SiO_2_-to-Al_2_O_3_ ratio in a zeolite (Ursini et al. 2006). According to Nibou et al. (2010), ion exchange can have one of two forms. On the one hand, the structure of channels in zeolite can be a factor reducing the exchange rate; on the other, the open structure of minerals ensures free ion exchange.

**Fig. 5:**
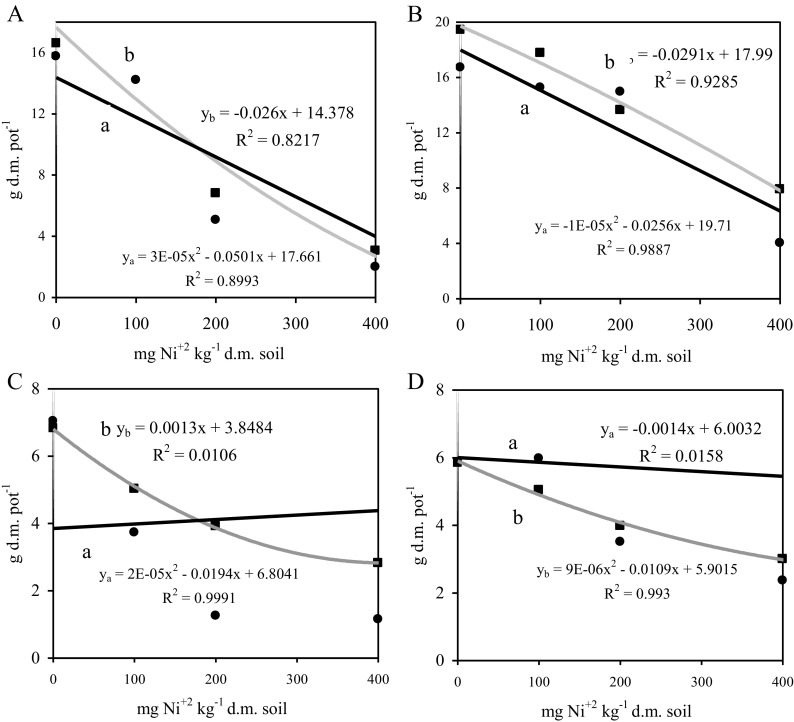
Yield of above-ground parts and roots (g d.m. pot^−1^) of oats from soil contaminated with nickel with an addition of zeolite. **a** loamy sand, above-ground parts; **b** silty-sandy loam, above-ground parts; **c** loamy sand, roots; **d** silty-sandy loam, roots. (a) control; (b) soil with an addition of zeolite

The percentage effect of variable factors indicates the greatest effect of the dose of the metal on oat yield: above-ground parts—77.49%, roots—67.97% (Table 4). The yield of the above-ground parts decreased with increasing contamination of soil with nickel, both in loamy sand and in sandy-silty loam (Fig. 5). The zeolite applied to the soil alleviated the negative impact on growth and development of plants only to a small extent. The effect of zeolite was significant (11.69%) only in the case of above-ground parts. A higher yield of the above-ground parts was obtained from the plants grown on sandy-silty loam and the yield of roots was similar in both soils. The content of nickel in the above-ground and in roots of oats as well as in soil increased with an increasing dose of nickel (Table 5). The plants grown on loamy sand were found to contain a larger concentration of the metal in the above-ground parts and roots than those grown on sandy-silty loam. An addition of zeolite Bio.Zeo.S.01 to loamy sand and to sandy-silty loam previously contaminated with nickel reduced the content of the metal in the above-ground parts and in roots of oats. The effect of the zeolite on nickel contents in above-ground parts and in roots depended on the granulometric composition of the soil. Better effects were observed for loamy sand. This may result from the fact that the loamy fraction in soils is more strongly affected by the volume of phytoavailable forms of heavy metals compared to the total content of heavy metals in soils and, consequently, has an effect on uptake and accumulation of these elements in plants (Zhou et al. 2014). Soils of high sorptive capacity, with a high content of dusty and silty formations and a high content of organic substance, are able to strongly bind heavy metals. Furthermore, acidic sandy soils of low sorptive capacity bind heavy metals poorly, which contributes to leaching of the elements from soil and their washing out to underground and surface waters (Muhammad et al. 2012).

**Table 4:** Percentage of factors of observed variability *η*^2^

Variable factors	Above-ground parts	Roots
Dose Ni^2+^ (a)	77.49	67.97
Type of soil (b)	11.69	0.79
Addition of zeolite (c)	1.09	0.90
a b	4.69	2.15
a c	0.40	1.99
b c	0.11	4.60
a b c	0.97	2.00
Error	3.56	19.60

**Table 5:** The content of nickel (mg kg^−1^ d.m.) in the above-ground parts and in roots of oats and in soil

Dose Ni^2+^ mg kg^−1^ of soil	Type of soil		
	Above-ground parts	Roots	Soil
Loamy sand			
Without the addition of zeolite			
0	9.20	24.00	7.10
200	72.00	169.20	107.20
$\overline{X}$	40.60	96.60	57.15
Zeolite Bio.Zeo.S.01			
0	8.80	43.00	11.60
200	35.20	120.00	87.40
$\overline{X}$	22.00	81.50	49.50
Silty-sandy loam			
Without the addition of zeolite			
0	3.90	31.00	18.50
200	57.60	77.40	108.40
$\overline{X}$	30.75	54.20	63.45
Zeolite Bio.Zeo.S.01			
0	3.80	70.00	18.20
200	43.00	85.20	120.85
$\overline{X}$	40.50	77.60	69.53

**Fig. 6:**
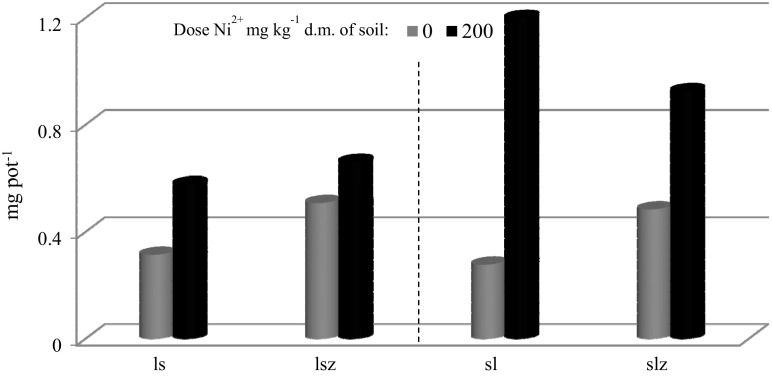
Absorption of nickel by oats (roots and above-ground parts), mg pot^−1^. ls, loamy sand; sl, silty-sandy loam; z, soil with an addition of zeolite

**Fig. 7:**
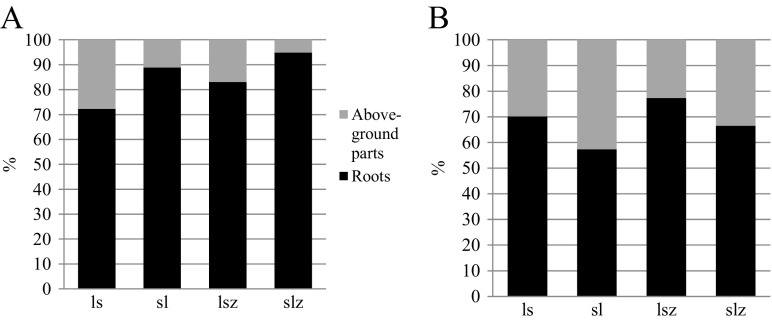
Redistribution of nickel in above-ground parts and in roots of oats, %. **a** soil non-contaminated with nickel; **b** soil contaminated with nickel (200 mg Ni^2+^ kg^−1^ d.m. of soil). ls, loamy sand; sl, silty-sandy loam; z, soil with an addition of zeolite

Absorption of nickel by oats increased with the dose of the metal applied (Fig. 6). The plants grown on sandy-silty loam were found to contain a larger concentration of nickel in the above-ground parts and roots than those grown on loamy sand. An addition of zeolite Bio.Zeo.S.01 to sandy-silty loam contaminated with nickel at 200 mg kg^−1^ d.m. of soil reduced the absorption of nickel by oats by 21%. This relationship was not observed in loamy sand. Figure 7 shows the distribution of nickel in above-ground parts and in roots. Larger amounts of nickel were absorbed by oat roots than by its above-ground parts, in soil both contaminated and non-contaminated by nickel. An addition of zeolite reduced the metal absorption by the above-ground parts in loamy sand and by roots in sandy-silty loam. The bioconcentration index (BAC) was calculated, taking into account the content of nickel in the above-ground parts of oats and in roots. The lowest value of this index was found in sandy-silty loam. The highest value of the BAC index was recorded in loamy sand and in sandy-silty loam contaminated with nickel and supplemented with zeolite (Table 6). These findings can be explained by the fact that the metal is rather easily absorbed by plants. Taken up by roots, it forms an anion complex and is rapidly transported to above-ground parts of a plant and is accumulated in vegetative and generative parts (Li et al. 2011; Rascio and Navari-Izzo 2011).

**Table 6:** Index of nickel bioconcentration (BAC)

Type of soil	Dose Ni^2+^ (mg kg^−1^ d.m. soil)	Zeolit Bio.Zeo.S.01 (g kg^−1^ d.m. soil)	Index BAC
ls	0	0	0.38
	200		0.43
ls	0	10	0.13
	200		0.74
sl	0	0	0.20
	200		0.29
sl	0	10	0.05
	200		0.50

*ls* loamy sand, *sl* silty-sandy loam

Physicochemical properties of the soils were also determined in the study (Table 7). Regardless of the addition of zeolite and the soil type, an addition of nickel decreased the pH and increased the hydrolytic acidity. These two parameters had a decisive effect on the sum of exchangeable cations. Contamination of soil with nickel only slightly modified the content of organic carbon, which remained at the same level in soils both with and without zeolite. The content of organic carbon was affected by the type of soil. It was higher in sandy-silty loam than in loamy sand. The findings are partly confirmed by literature reports, because heavy metals, including nickel, can acidify soils, but the extent of their effect on soil properties depends mainly on its type and level of acidification (Hooda 2010; Muhammad et al. 2012). A number of heavy metals are better soluble and biologically available to plants at lower pH (5.4–6.6) than at higher levels (7.8–8.2), according to a report by Hooda (2010). Muhammad et al. (2012) also stress that pH of soil has a significant effect on the amount of available heavy metal species in soil and the bioavailability of elements increases with its decreasing level. This results in their contamination and including heavy metals in the food chain of other organisms (Hooda 2010).

**Table 7:** Physicochemical properties of soil with an addition of zeolite and contaminated with nickel

Dose Ni^2+^ (mg kg^−1^ d.m. soil)	pH_KCl_	C_org_ g kg^−1^	HAC	EBC	CEC	
			mmol^(+)^ kg^−1^ of soil			BS %
Loamy sand						
Without the addition of zeolite						
0	7.30	9.44	8.63	85.00	93.63	90.77
100	7.20	9.28	8.63	82.50	91.13	90.53
200	7.00	9.22	9.00	80.50	89.50	89.94
400	6.90	8.85	9.95	75.50	85.45	88.30
$\overline{X}$	7.10	9.20	9.05	80.88	89.93	89.89
r*	− 0.96	− 0.99	0.96	− 0.99	− 0.99	− 0.98
Zeolit Bio.Zeo.S.01						
0	7.10	9.09	8.63	78.50	87.13	90.08
100	7.00	8.81	8.63	75.50	84.13	89.74
200	6.85	8.27	10.50	71.50	82.00	87.19
400	6.75	8.24	11.08	71.00	82.08	86.51
$\overline{X}$	6.93	8.60	9.71	74.13	83.84	88.38
r*	− 0.97	− 0.89	0.92	− 0.90	− 0.84	− 0.92
Silty-sandy loam						
Without the addition of zeolite						
0	7.40	14.36	6.58	117.50	124.08	94.70
100	7.40	13.67	6.95	117.00	123.95	94.38
200	7.30	13.66	7.13	114.50	121.63	94.14
400	7.20	13.53	8.83	107.50	116.33	92.41
$\overline{X}$	7.33	13.81	7.37	114.13	121.50	93.91
r*	− 0.97	− 0.79	0.96	− 0.97	− 0.97	− 0.96
Zeolit Bio.Zeo.S.01						
0	7.40	14.50	6.58	120.00	126.58	94.80
100	7.30	13.74	7.50	119.50	127.00	94.09
200	7.20	13.64	9.20	117.50	126.70	92.74
400	7.10	12.82	9.95	104.50	114.45	91.30
$\overline{X}$	7.25	13.68	8.31	115.38	123.68	93.23
r*	− 0.98	− 0.97	0.96	− 0.94	− 0.87	− 0.99

*ls* loamy sand, *sl* silty-sandy loam, *C*_*org*_ total organic carbon, *HAC* hydrolytic acidity, *EBC* total exchangeable cations, *CEC* total exchange capacity of soil, *BS* basic cations saturation ratio in soil

## Conclusions

The activity of dehydrogenases, catalase, urease, acid phosphatase, alkaline phosphatase, β-glucosidase and arylsulfatase decreased significantly in soil contaminated with nickel. The activity of all of the enzymes was higher in sandy-silty loam than in loamy sand. The growth and development of oats were significantly disrupted by an excess of nickel in the soil. An addition of zeolite decreased the level of accumulation of nickel in oats grown only on sandy-silty loam. The metal was accumulated more in the oat roots than in the above-ground parts. Zeolite Bio.Zeo.S.01 only slightly alleviated a negative effect of nickel on the biochemical properties of soil. Therefore, its use in the remediation of soil contaminated with nickel is small.
